# Natural Barcodes for Longitudinal Single Cell Tracking of Leukemic and Immune Cell Dynamics

**DOI:** 10.3389/fimmu.2021.788891

**Published:** 2022-01-03

**Authors:** Livius Penter, Satyen H. Gohil, Catherine J. Wu

**Affiliations:** ^1^ Department of Medical Oncology, Dana-Farber Cancer Institute, Boston, MA, United States; ^2^ Broad Institute of Massachusetts Institute of Technology and Harvard University, Cambridge, MA, United States; ^3^ Harvard Medical School, Boston, MA, United States; ^4^ Department of Hematology, Oncology, and Tumorimmunology, Campus Virchow Klinikum, Berlin, Charité – Universitätsmedizin Berlin, Corporate Member of Freie Universität Berlin and Humboldt-Universität zu Berlin, Berlin, Germany; ^5^ Department of Academic Haematology, University College London Cancer Institute, London, United Kingdom; ^6^ Department of Haematology, University College London Hospitals NHS Foundation Trust, London, United Kingdom; ^7^ Department of Medicine, Brigham and Women’s Hospital, Boston, MA, United States

**Keywords:** copy number variants (CNV), somatic nuclear mutation, mitochondrial DNA mutation, single nucleotide polymorphism, B cell receptor sequence, T cell receptor sequence, allogeneic hematopoietic stem cell transplantation (allo-HCT), single-cell sequencing

## Abstract

Blood malignancies provide unique opportunities for longitudinal tracking of disease evolution following therapeutic bottlenecks and for the monitoring of changes in anti-tumor immunity. The expanding development of multi-modal single-cell sequencing technologies affords newer platforms to elucidate the mechanisms underlying these processes at unprecedented resolution. Furthermore, the identification of molecular events that can serve as *in-vivo* barcodes now facilitate the tracking of the trajectories of malignant and of immune cell populations over time within primary human samples, as these permit unambiguous identification of the clonal lineage of cell populations within heterogeneous phenotypes. Here, we provide an overview of the potential for chromosomal copy number changes, somatic nuclear and mitochondrial DNA mutations, single nucleotide polymorphisms, and T and B cell receptor sequences to serve as personal natural barcodes and review technical implementations in single-cell analysis workflows. Applications of these methodologies include the study of acquired therapeutic resistance and the dissection of donor- and host cellular interactions in the context of allogeneic hematopoietic stem cell transplantation.

## Introduction

After decades of research, cancer has remained a formidable enemy with relapse as an all too frequent outcome despite advances in treatment approaches. Deeper elucidation of the underlying malignant cell states and reprogrammed immune circuits will help to overcome current therapeutic limitations and improve long-term outcomes. Single-cell sequencing technologies are providing capabilities to understand cellular states at unprecedented depth, which has greatly accelerated our understanding of hematopoiesis (1–7) and haematological malignancies (8–14). Certainly, the dense cellular sampling of biospecimens, afforded by single-cell epigenomics and transcriptomics, has allowed the inference of trajectories of cell differentiation from primary human samples and has deepened our understanding of the disruption between physiologic and malignant states (15).

A growing area of interest is the use of molecular barcodes to corroborate these insights experimentally. Technologies that introduce artificial barcodes such as fluorescence-based labeling, viral barcoding, Cre-Lox-based approaches and CRISPR-Cas9 genome editing enable prospective lineage tracing (16, 17) and permit the characterization of the phenotype of defined genotypes in great detail (18, 19). However, these studies are limited by the experimental models they employ. In addition, the introduction of barcodes may lead to unintended perturbation of cell states and off-target effects, thus skewing the studied phenotypes.

An alternative approach is to leverage naturally occurring molecular barcodes to perform retrospective *in-vivo* lineage tracing in unaltered primary human cells. This approach has already proven to be a powerful strategy for deconvoluting clonal cancer fractions from bulk sequencing data through the analysis of evolving variant allele frequencies of single nucleotide variants (20, 21). When coupled with single-cell analyses, the use of natural barcodes provides the potential for delineating the phylogeny of cell populations at much greater resolution, and depending on the technology may link the genotypes of individual cells to distinct phenotypic cell states (22).

Here we review the growing number of single-cell methodologies for exploiting such natural barcodes. As examples, we describe the utility of T or B cell receptor (TCR, BCR) sequences and copy number changes, somatic nuclear and mitochondrial DNA (mtDNA) mutations for lineage tracing. We further discuss specific opportunities in the setting of allogeneic hematopoietic stem cell transplantation (HSCT).

## Tool Kits for Longitudinal Tracking of Disease Evolution at the Single-Cell Level

Efforts to utilize natural barcodes to elucidate the developmental history of individual cells have preceded the availability of current single-cell sequencing modalities. Despite their low throughput, these early attempts laid the groundwork for our understanding how allelic variants can serve to track cell populations longitudinally over months or years at single-cell resolution. They have included techniques such as red blood cell phenotyping (23, 24), fluorescence *in-situ* hybridization (FISH) using probes specific for X and Y chromosomes and cell type-specific staining (25, 26), analysis of sex-linked electrophoretic variants of glucose-6-phosphate dehydrogenase (27) or clinical karyotyping (28). Although these techniques are amenable to single-cell sequencing approaches (29), they are mostly constrained to specific contexts that limits their wider applicability.

### Physiologic Natural Barcodes

**Figure 1:**
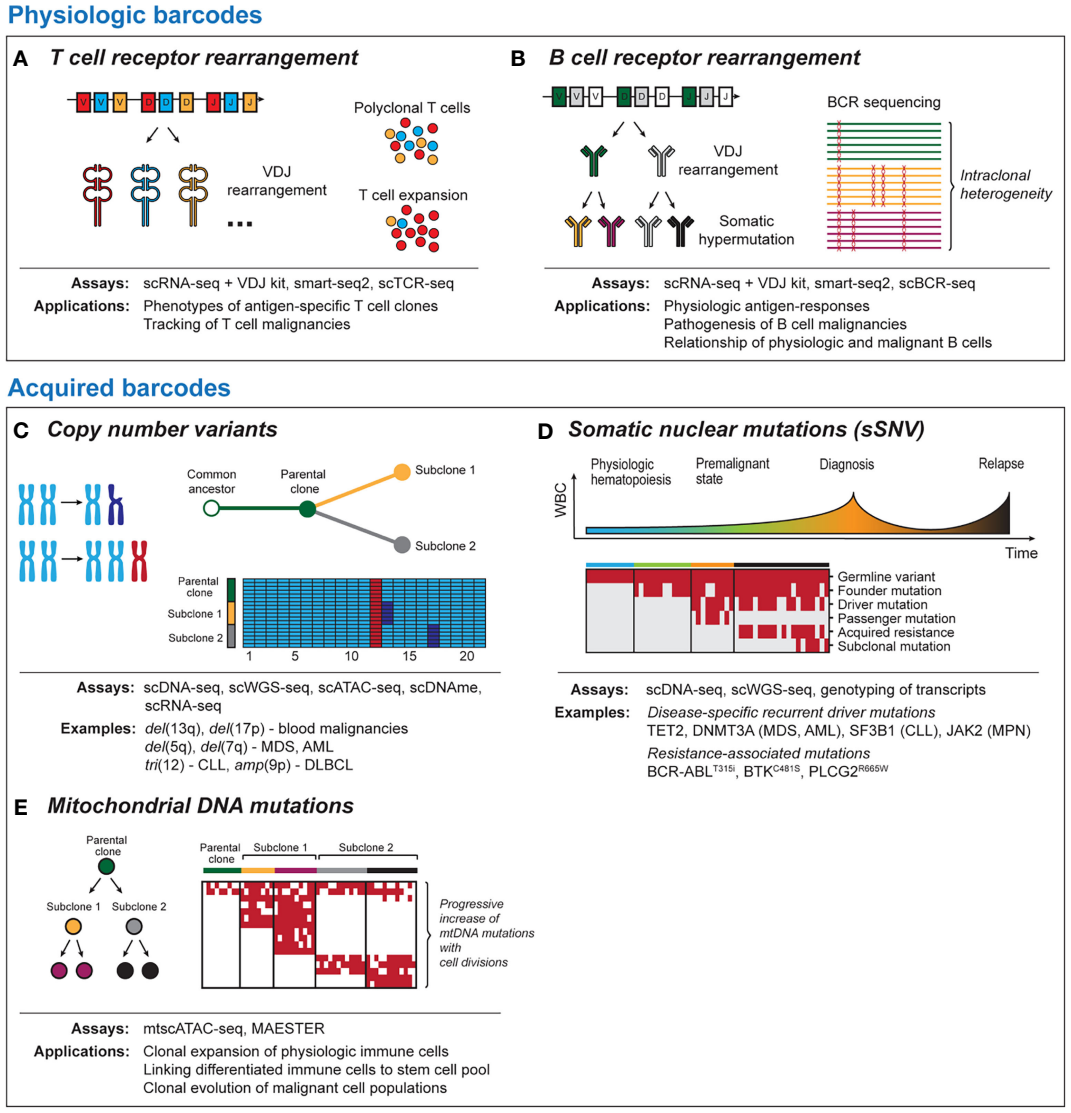
Tool kits for lineage tracing with single-cell sequencing. Physiologic barcodes **(A)** T cell receptor (TCR) sequencing detects clonal expansion of T cells based on VDJ rearrangement and junctional diversification. TCR can help to identify and phenotype antigen-specific T cells or track malignant T cells. **(B)** B cell receptor (BCR) sequences arise due to VDJ rearrangement and ongoing somatic hypermutation. BCR can provide insight into physiological antigen responses and intraclonal heterogeneity in post-germinal B cell malignancies. Both BCR and TCR are best read out with RNA-based single-cell platforms due to the high number of BCR/TCR mRNA templates per cell. Acquired barcodes **(C)** Chromosomal copy number variants (CNV) are common aberrations in blood malignancies. CNV can provide robust signals with DNA- and RNA-based sequencing platforms that allow to dissect subclonal structure of cancer and can be detected using most single-cell sequencing platforms. **(D)** Somatic nuclear mutations (single somatic-nucleotide variant, sSNV) can track clonal evolution in cancer longitudinally. Different classes of somatic mutations are distinguished such as germline variants, cancer initiating mutations, mutations associated with therapeutic resistance and sporadic mutations unrelated to the disease pathogenesis. **(E)** Mitochondrial DNA mutations are progressively acquired as cells divide. This allows to link physiologic or malignant cells to a common ancestor and to resolve phylogeny. As coverage of mitochondrial transcripts tends to be incomplete, they can be best read out from DNA-based sequencing platforms. scDNA-seq, single-cell DNA sequencing; scWGS-seq, single-cell whole genome sequencing; scATAC-seq, single-cell Assay for Transposase-Accessible Chromatin with high-throughput sequencing; scDNAme, single-cell sequencing of DNA methylation; scRNA-seq, single-cell RNA sequencing; mtscATAC-seq, mitochondrial scATAC-seq; scBCR-seq, single-cell B cell receptor sequencing; scTCR-seq, single-cell T cell receptor sequencing.

The TCR and BCR are examples of physiologic molecules with high diversity which lend themselves as natural barcodes that can be used for lineage tracing purposes (**Figure 1A**). Each unique TCR arises from a combination of α/β or γ/δ chains with highly variable sequences due to V(D)J rearrangement and junctional diversification (30). Single-cell sequencing of TCR now enables phenotyping of individual T cell clones (31, 32) and permits tracking of antigen-specific T cells across tissue compartments and following therapeutic interventions such as immune checkpoint blockade or vaccination (33–35). Further, TCR is used to phenotype malignant T cells, for example in angioimmunoblastic (36) or cutaneous T cell lymphoma (37, 38).

Compared to T cells, B cells additionally undergo affinity maturation through somatic hypermutation (SHM), which renders the BCR repertoire even more dynamic (**Figure 1B**) (39, 40). Besides applications to understanding immunoglobulin responses to viral or tumor-specific antigens (41–43), the ongoing changes in BCR enable lineage tracing of post-germinal center B cell malignancies that have undergone V(D)J rearrangement and SHM (44). Sequencing of follicular lymphoma (45), DLBCL (46) and multiple myeloma (47) has demonstrated intraclonal BCR heterogeneity that can shed light on clonal evolution following malignant transformation. Further, BCR sequences can be used to dissect disease pathogenesis (48, 49) or mechanisms underlying differential therapeutic sensitivity (50) of B cell malignancies.

Several RNA-based single-cell TCR/BCR sequencing platforms exist that differ in the length of covered sequence and error rate. 5’ short read droplet-based sequencing yields CDR3 sequences by amplifying cDNA using primers specific for TCR and immunoglobulin constant regions, but is unable to cover most of the V region. This approach currently has the highest throughput and can resolve phenotypes of TCR/BCR clonotypes with detailed resolution. Smart-seq2 with computational reconstruction of TCR/BCR sequences leads to better coverage, but has a higher cost per sample and lower throughput (51–53). These limitations are being improved with the development of smart-seq3 (54, 55). Long-read sequencing can provide full-length TCR/BCR sequences in a large number of cells, although with higher error rates (56, 57). These technologies are therefore suited for studies that require information on regions outside the CDR3 sequence. Finally, cost-effective targeted approaches with multiplexed PCR can be an option for analysis of rare cells or little starting material (47, 58). Different analysis pipelines for these platforms have been developed that include cellranger (5’ scRNA-seq with VD(J) enrichment) (59), MiXCR (60) or TRUST4 (61).

### Copy Number Changes

Chromosomal copy number variations (CNV) can provide robust signals that are detectable with RNA- and DNA-based sequencing platforms (**Figure 1C**). The high prevalence of CNV changes in blood malignancies and the genomic instability associated with relapse following therapy (62–65) render them as useful barcodes for lineage tracing and phylogenetic dissection of subclones within malignant cell populations. Examples of recurrent CNV events include *del*(5q) and *del*(7q) in myeloid disease or *tri*(12) and *del*(17p) in CLL. A strength of lineage tracing based on CNV changes is the shallow sequencing depth required for detection (66), which allows greater numbers of cells to be analyzed. Limitations for the utility of CNV changes for lineage tracing are either the common absence of chromosomal aberrations as in diseases such as AML with normal karyotype (67) or their omnipresence within monoclonal populations, if they are early founding events.

DNA-based single-cell assays can detect CNV in targeted regions of interest (scDNA-seq) or globally across the entire nuclear genome [e.g. single-cell whole genome (scWGS-seq) (68–70), assay for transposase-accessible chromatin using sequencing (scATAC-seq) (71, 72) or single-cell sequencing of DNA methylation (scDNAme) (73)]. Targeted approaches enable detection of CNV changes at higher coverage and better cost efficiency, but require design of primers for these target regions, which can be accomplished either through large panels for recurrent genetic events or personalized solutions based on previous analyses (9, 74). While scDNA-seq and scWGS-seq profiles are unable to natively identify cell types and thus require either purification of cell populations prior to sequencing or additional detection of cell surface marker expression, scATAC-seq and scDNAme have the advantage of providing combined information on CNV and cell state, for example through capture of accessible chromatin (75). A major technical hurdle is the fact that detection of subchromosomal CNV is prone to false positive calls due to effects such as unequal coverage of genomic regions or PCR amplification bias. To address this, several computational methods have been developed to overcome these challenges (72, 76, 77).

For transcriptomic data, the tool inferCNV, which is based on comparison of read coverage between target and reference cell types, has gained wide usage (78, 79). The main limitations of inference of CNV from transcriptomic data are cell type-specific expression profiles and the unequal gene coverage biased towards the 3’ or 5’ end, which can lead to false-positive results, especially for smaller CNV regions (80). It is therefore recommended to establish ground truth knowledge of CNV changes using orthogonal technologies such as whole exome sequencing (WES) or clinical karyotyping. Other tools for inferring CNV from single-cell transcriptomes include HoneyBADGER (81), scCNAutils (82), CaSpER (83), DENDRO (84) or CopyKAT (85).

### Somatic Nuclear DNA Mutations

Together with CNV, single somatic-nucleotide variants (sSNVs) of nuclear DNA have been at the center of numerous efforts to understand cancer evolution using bulk sequencing approaches (86). Although somatic mutations are less frequent in blood malignancies compared to solid tumors (87), they often can be linked to altered gene function implicated in tumorigenesis such as mutations in *TET2* (88), *DNMT3A* (89) (MDS/AML), *SF3B1* (90, 91) (CLL, MDS) or *JAK2 (* 92) (MPN) and acquired therapeutic resistance (BCR-ABL^T315i^, BTK^C481S^, PLCG2^R665W^) (93, 94) (**Figure 1D**). While bulk sequencing infers clonal structures bioinformatically, single cell sequencing can directly measure coexistence of mutations within individual cells. Currently, two major strategies for identification of somatic mutations exist. These include either targeted scDNA-seq with a primary focus on variant calling or genotyping of single cell transcriptomics. The advantage of scDNA-seq is the ability to genotype dozens of loci in thousands of single cells, which provides very high resolution for tracking of mutation dynamics across cell compartments and time. RNA-based approaches are less efficient but can establish links between altered cell states and somatic mutations, for example through deconvolution of differential gene expression between mutated and non-mutated cells within the same cluster (8, 95). Importantly, both approaches are prone to allelic dropout, an inherent limitation of PCR amplification from limited starting material (96).

For targeted scDNA-seq platforms, primer panels for recurrent somatic mutations facilitate identification of these disease-specific variants (9, 22, 97). However, this approach is unable to target personal mutations and less frequent variants (98, 99) that would otherwise substantially increase the resolution of leukemic evolution (100, 101). This shortcoming can be addressed either through screening of personal mutations with WES and subsequent targeted single-cell sequencing (102, 103), or with unbiased scWGS-seq. Similar to the analysis of CNV, a critical step in the identification of somatic nuclear mutations from scWGS-seq involves stringent filtering of false positive results (104). Mutation calling can be performed with the Tapestri pipeline, which is based on the variant caller GATK4 (105).

The calling of somatic nuclear mutations from short-read transcriptomic sequencing libraries is conceptually feasible but in actuality faces several challenges. Due to the 3’ or 5’ bias and shallow coverage of individual transcripts, somatic mutations can only be reliably called in a minority of cells (<5%) from unmodified single-cell gene expression profiles. Targeted amplification of regions of interest can increase the coverage of mutated loci and enables mutation calling in a higher percentage of cells, ranging from <10 to >50% depending on the underlying sequencing platform, such as 3’ or 5’ bias for short-read sequencing, the location of the mutation within the transcript and its expression level (8, 75, 106). For processing of amplicons, custom solutions have been developed (8, 95). At the present time, this approach works best for loci close to the end of the cDNA template, while for mutations that are more distant, long-read sequencing can increase their coverage (56, 95).

### Mitochondrial DNA Mutations

While CNV and somatic nuclear mutations are well-established approaches to lineage tracing, recently it has been recognized that mitochondrial DNA (mtDNA) mutations have the potential to serve as phylogenetic barcodes (**Figure 1E**) (107, 108). This provides opportunities for lineage-tracing at increased resolution within cell populations defined by the same set of somatic mutations or even in absence of such genetic events. Compared to somatic nuclear mutations, mtDNA mutations have various advantages. Mitochondria can replicate independently of the cell cycle, and thus mtDNA may be present in hundreds of copies per cell. In combination with the small size (~16.6 kB), mtDNA can therefore be sequenced with high coverage at single-cell resolution. Finally, mtDNA has a considerably higher mutation rate compared to genomic DNA (109, 110).

In principle, mtDNA mutation can be read out with the same approaches that apply to somatic nuclear mutations, however due to the unique biology of mtDNA, specific tools are being developed: mtscATAC-seq is a multi-omics platform that provides the feasibility of integrating information on cell state, CNV and mtDNA mutations of the entire mtDNA genome at single-cell resolution. For these studies, mgatk is an optimized variant caller which utilizes strand concordance of forward and reverse reads as well as mean variance ratio to enrich for true-positive mtDNA mutations (77).

The detection of mtDNA mutations from single-cell transcriptomic profiles is challenging as the coverage of mitochondrial transcripts is typically heterogeneous and insufficient to call mutations for large parts of the mtDNA genome, with a tendency for better coverage using full-length RNA-sequencing (108, 111). The MAESTER protocol overcomes these hurdles through a 65-primer multiplexed PCR amplification of mitochondrial transcripts to achieve sufficient coverage for mutation calling (112). By excluding UMIs with fewer than 3 reads and through consensus calling of reads from the same UMI, the variant caller optimized for MAESTER (maegtk) reduces false-positive mtDNA mutations deriving from PCR errors, and paves the way for more consistent calling of mtDNA mutations.

While studies on mtDNA mutations in blood malignancies are still in their infancy, it is already clear that they (i) are able to define subclonal structure within monoclonal cell populations, (ii) remain stable over time in absence of strong selective pressure and (iii) change in frequency following therapeutic bottlenecks (75, 77, 106). Further, there is evidence that mtDNA and somatic nuclear mutations provide complementary information on clonal evolution in leukemia (106). However, many open questions remain such as whether mtDNA mutations provide selective advantage for example through alteration of oxidative phosphorylation (113, 114) or whether they are sufficiently sensitive and specific for the tracking of malignant clones in the context of low disease burden such as minimal residual disease.

In the non-malignant context, mtDNA mutations offer a possibility to track lineage within cell populations that lack natural barcodes such as monocytes or natural killer cells and enable to link differentiated cells to their progenitor and stem cell populations (115). There are also examples of how mtDNA mutations can track clonal expansion of T cells and further subdivide T cells with the same T cell receptor sequence, potentially providing an avenue to dissect dynamics within a T cell clone (75, 108).

## Lineage-Tracing in the Context of Allogeneic Stem Cell Transplantation

**Figure 2:**
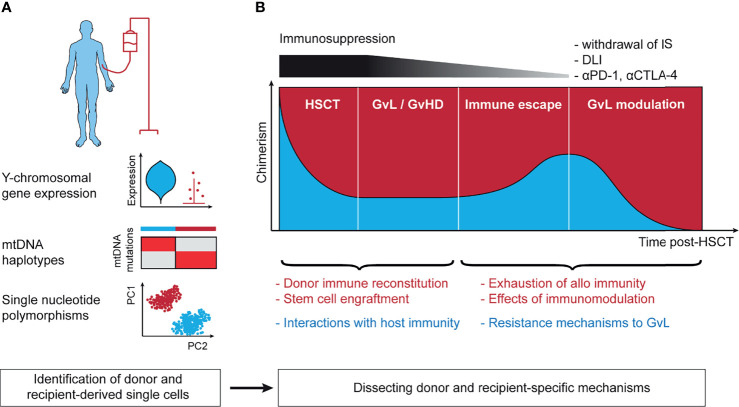
Lineage-tracing in the context of allogeneic hematopoietic stem cell transplantation. **(A)** Technical approaches to annotation of donor- and recipient-derived cells. Y chromosomal gene expression is able to robustly separate donor and recipient in the context of sex-mismatched transplantation. mtDNA haplotypes leverage germline single nucleotide polymorphisms in the mitochondrial genome and can distinguish between matched-unrelated donor and recipient. Single nucleotide polymorphisms distinguish between all donor and recipient pairs except for identical twins. **(B)** The post-transplant setting harbors context-specific questions in the 4 stages following stem cell infusion. These include (1) the mechanisms of initial stem cell engraftment, (2) the interaction of host and recipient as basis for GvL and GvHD, (3) immune escape leading to disease relapse, and (4) reinvigoration of GvL following effective immunotherapeutic intervention. mtDNA, mitochondrial DNA; IS, immunosuppression; DLI, donor-lymphocyte infusion; αPD-1, antibody against programmed cell death protein 1 (PD-1); αCTLA-4, antibody against cytotoxic T-lymphocyte-associated protein 4; HSCT, hematopoietic stem cell transplantation; GvL, graft-versus-leukemia; GvHD, graft-versus-host disease; PC1/2, principal component 1/2.

HSCT is arguably the longest standing and the most successful immunotherapy with the graft-versus-leukemia/lymphoma (GvL) effect at its core (116). Research into target antigens of GvL and processes such as donor reconstitution or immunomodulation post-HSCT have advanced our understanding of fundamental immune processes (117–119). Despite these insights, many questions remain unanswered such as the exact mechanisms of response to immune modulation post-HSCT like donor-lymphocyte infusion (DLI) or interactions between donor and host under conditions of mixed chimerism. Lineage-tracing approaches are an opportunity to address these questions, leveraging the coexistence of donor- and recipient-derived cells but also require robust annotation of these two populations (**Figure 2A**).

### Sex Mismatch

In the case of sex-mismatched HSCT (120), annotation of cells to donor and recipient can be based on expression of Y chromosomal genes including *RPS4Y1* and *DDX3Y* or genes implicated in X-inactivation such as *XIST* (121). As scRNA-seq data are typically sparse, with dropout of gene expression, higher accuracy can be expected from annotation using multiple genes. The strength of this approach is that no additional sequencing is needed and that it allows for annotation of even rare cells through a simple but robust analysis. Examples include tracking of circulating host tissue-resident T cells following myeloablative conditioning (122) or donor-derived immune cells following solid organ transplant (123). A limitation is that sex mismatch only occurs in a subset of transplants.

### Single Nucleotide Polymorphisms

Germline single nucleotide polymorphisms (SNP) can be used to distinguish donor and recipient unambiguously. Due to the large number of more than 1 million SNPs in the human genome (124), even siblings, except for identical twins, differ in their set of SNPs, thus allowing to identify the origin of single-cells from sparse scRNA-seq data with shallow coverage of individual loci. This can be achieved either through annotation with a genotype reference obtained using WES of purified donor or recipient-derived cells (125, 126) or with reference-free approaches that are based on statistical modeling. Tools that implement the latter strategy for scRNA-seq were originally developed for deconvolution of samples from different donors but can be used for assignment of donor and host at single-cell resolution (127–130). For scWGS-seq and scATAC-seq data, a similar approach is possible (131, 132), but has not been implemented yet.

### mtDNA Haplotypes

Reference-free SNP-based annotation of donor- and recipient-derived cells from sparse single-cell data is based on clustering of similar cells and therefore accurate annotation of rare cells can be challenging. A possible alternative is to utilize mtDNA haplotypes for this purpose that arise from SNPs in the mitochondrial genome (133). From a technical standpoint this approach has the advantage that owing to the small size of the mtDNA genome and its high density of SNPs, the mtDNA haplotype is very informative and allows to unambiguously annotate the cell origin even from sparse single-cell data. This can be achieved either using the mtscATAC-seq protocol or with targeted amplification from scRNA-seq libraries (see Section “*Mitochondrial DNA Mutations*”). The only limitation is that due to the matrilineal inheritance of mitochondria, this approach is mainly useable for transplants from matched-unrelated or certain haploidentical donors.

### Opportunities for Understanding HSCT With Single-Cell Sequencing

As a complex immunotherapy, there are many open questions regarding HSCT than can be divided by the 4 stages of the post-transplant setting: (1) engraftment (134) and immune reconstitution (135) following HSCT, (2) GvL and GvHD when stable engraftment has been achieved (136), (3) immune escape mechanisms leading to relapse (137), and (4) immune modulation approaches to reinstate effective GvL (117) (**Figure 2B**). Single-cell sequencing offers windows into gaining broad understanding of these processes in detail and can potentially leverage all lineage-tracing approaches discussed in this text. Although progress in these directions has been made (75, 100, 138), the envisioned efforts are still in their infancy.

Single-cell transcriptomics are currently underway to describe immune cell populations implicated in GvHD such as B cells (139), regulatory T cells (140), or cytotoxic CD4^+^ T cells (141). In the latter example, the authors tracked expansion of a mutation in the *mTOR* gene within donor-derived T cells which they associate with persistent immune activation and GvHD. Lineage-tracing approaches have the potential to deepen the understanding of GvHD by dissecting the contribution of donor and recipient-derived T cells to GvHD target organs. An example of this approach demonstrated that tissue-resident memory T cells can retain a large fraction of host cells despite full systemic chimerism (122).

More generally, conditions of mixed chimerism are an area where high-throughput distinguishing of donor and recipient will be able to address questions that so far have been difficult to answer, for example whether recipient-derived cells persist in specific T cell subsets such as CD8^+^ T cells of patients with aplastic anemia (142). Similarly, lineage-tracing will allow the understanding of the engraftment of hematopoietic stem cells in more detail, for example in the context of donor clonal hematopoiesis of indeterminate potential (CHIP) where somatic mutations can serve as barcodes (143).

Finally, mechanisms that underly disease relapse following HSCT (75, 100, 144) and response to therapies that reinstate GvL through DLI (145) or checkpoint immune blockade (119, 146) are increasingly characterized using single-cell sequencing. Lineage-tracing has many potential applications in these studies, for example finer dissection of intraclonal evolution (147) or tracking of exhausted T cells before and after immunomodulation, as has been demonstrated in a large-scale characterization of bone marrow-derived T cell states following effective DLI (145, 148).

## Discussion

*In-vivo* lineage tracing approaches using natural barcodes are seeing breathtaking progress and technological advances of single-cell sequencing are enabling studies that seemed inconceivable only a few years ago. While technical hurdles are being removed, new questions are becoming relevant. With multi-omics sequencing platforms pushing the boundaries of what is possible, these technologies are also associated with unprecedented costs per analyzed sample, thereby leading to focus on select samples. Undoubtedly, high-resolution and multi-modal analyses of individual samples are exciting, yet true advances will depend on applying these novel technologies on well-designed clinical cohorts with longitudinal sampling, for example in the setting of clinical trials that aim to answer specific biological questions. With the prospect of single-cell sequencing achieving hundreds of thousands of cells per sample throughput, clinical applications that leverage longitudinal lineage-tracing approaches such as the measuring of minimal residual disease become a possibility.

## Author Contributions

LP designed the figures and wrote the manuscript. SG provided critical input. CW supervised and edited the manuscript. All authors contributed to the article and approved the submitted version.

## Funding

This work was supported by grants from the National Cancer Institute (UG1 CA233338, 1U24CA224331-01, and P01CA229092 to CW). LP is supported by a research fellowship from the German Research Foundation (DFG, PE 3127/1-1). SG is supported by a Kay Kendall Leukaemia Fund Fellowship.

## Conflict of Interest

CW holds equity in BioNTech,Inc; and receives research support from Pharmacyclics. SG undertakes consultancy for Novalgen Limited and has received speakers fees and honoraria from Abbvie, Janssen and AstraZeneca.

The remaining author declares that the research was conducted in the absence of any commercial or financial relationships that could be construed as a potential conflict of interest.

## Publisher’s Note

All claims expressed in this article are solely those of the authors and do not necessarily represent those of their affiliated organizations, or those of the publisher, the editors and the reviewers. Any product that may be evaluated in this article, or claim that may be made by its manufacturer, is not guaranteed or endorsed by the publisher.
